# Three-dimensional fluorescence imaging using the transport of intensity equation

**DOI:** 10.1117/1.JBO.25.3.032004

**Published:** 2019-11-12

**Authors:** Sudheesh K. Rajput, Manoj Kumar, Xiangyu Quan, Mitsuhiro Morita, Tomoyuki Furuyashiki, Yasuhiro Awatsuji, Enrique Tajahuerce, Osamu Matoba

**Affiliations:** a Kobe University, Graduate School of System Informatics, Department of Systems Science, Nada, Kobe, Japan; b Kobe University, Graduate School of Sciences, Department of Biology, Nada, Kobe, Japan; c Kobe University, Graduate School of Medicine, Division of Pharmacology, Chuo-ku, Kobe, Japan; d AMED-CREST, Chiyoda-ku, Tokyo, Japan; e Kyoto Institute of Technology, Faculty of Electrical Engineering and Electronics, Matsugasaki, Sakyo-ku, Kyoto, Japan; f Universitat Jaume I, Institute of New Imaging Technologies (INIT), Department of Physics, Castello, Spain

**Keywords:** 3-D fluorescence imaging, transport of intensity equation, phase retrieval, Fresnel propagation

## Abstract

We propose a nonscanning three-dimensional (3-D) fluorescence imaging technique using the transport of intensity equation (TIE) and free-space Fresnel propagation. In this imaging technique, a phase distribution corresponding to defocused fluorescence images with a point-light-source-like shape is retrieved by a TIE-based phase retrieval algorithm. From the obtained phase distribution, and its corresponding amplitude distribution, of the defocused fluorescence image, various images at different distances can be reconstructed at the desired plane after Fresnel propagation of the complex wave function. Through the proposed imaging approach, the 3-D fluorescence imaging can be performed in multiple planes. The fluorescence intensity images are captured with the help of an electrically tunable lens; hence, the imaging technique is free from motion artifacts. We present experimental results corresponding to microbeads and a biological sample to demonstrate the proposed 3-D fluorescence imaging technique.

## 1 Introduction

Fluorescence imaging is an important technique to get the functional information of a biological sample for cellular and microbiological investigations, as has been proved by several studies.^1^^–^^13^ Most of the reported fluorescence imaging techniques are either two-dimensional in nature or involve sectioning to get three-dimensional (3-D) information, such as laser scanning confocal microscopy and other related techniques.^1^^–^^11^ These techniques are time-consuming processes to obtain the 3-D information of objects. Further techniques have been developed by using digital holography in fluorescence microscopy to record and retrieve the 3-D information of a fluorescent object.^12^^,^^13^ However, by adopting digital holography or other interferometric systems for 3-D fluorescence imaging, the imaging systems become more complicated due to the involvement of an additional reference beam. So, it is desirable to further investigate 3-D fluorescence imaging techniques to develop simple, compact, and cost-effective solutions.

Noninterferometric imaging techniques, such as those based on the transport of intensity equation (TIE), have been proposed for quantitative phase imaging,^14^^–^^22^ which does not require a reference beam and hence makes the system simple and compact. Teague proposed the TIE from the Helmholtz equation under the paraxial approximation with the assumption of a monochromatic and coherent beam.^14^ Some reported variants of the TIE method work with partial coherence illumination.^15^^–^^20^ Hence, the TIE technique has an additional advantage of robustness against disturbance under the partially coherent illumination, which extends its application to microscopy.^17^^–^^22^ In these techniques, it is often inevitable or desirable that the field should be partially coherent. Generally, the TIE method requires two or three intensity measurements recorded at different planes to retrieve the phase distribution. In addition to simple optical phase microscopy, it has been demonstrated that accurate and high-quality quantitative phase imaging can be achieved by imaging techniques based on TIE with partially coherent illuminations to prevent image degradation due to speckle noise.^15^^–^^20^ Focus estimation has also been proposed in TIE phase imaging using inverse Fresnel propagation.^21^^,^^22^ To apply this concept in fluorescence imaging could be advantageous in different applications requiring 3-D fluorescence imaging.

Here, we propose the use of the TIE for the 3-D fluorescence imaging. With the help of a TIE-based phase retrieval algorithm and Fresnel backpropagation, images focused at different distances can be obtained from three defocused fluorescent images recorded in multiple planes. To the best of our knowledge, this is the first paper to demonstrate 3-D fluorescence imaging of biological samples by TIE. Although fluorescence light is incoherent, the partial spatial coherence of defocused images obtained by propagation is available when specific conditions, such as small fluorescence light sources (from nuclei of the cells) and narrow bandwidth, are assumed. Note that the phase distribution is measured in the image defocused plane, not in the object plane where the nuclei of the cells exist. The proposed imaging system is simple and straightforward and can be used for different kinds of fluorescent samples. The idea is demonstrated experimentally by developing a specific 3-D microscopy optical setup.

## 2 Methodology

**Fig. 1:**
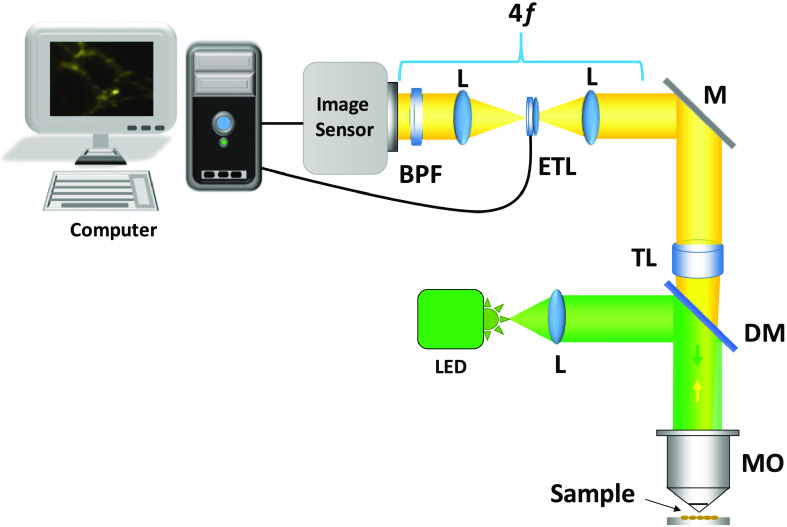
The 3-D fluorescence imaging setup. LED, light-emitting diode; L, lens; TL, tube lens; ETL, electrically tunable lens; MO, microscope objective; M, mirror; DM, dichroic mirror; and BPF, bandpass filter.

The optical setup of the proposed 3-D fluorescence imaging system is shown in Fig. 1. In this system, a blue LED light source was used to excite the sample placed on the stage. Three defocus intensity images were recorded by changing the optical power of the commercially available electrically tunable lens (ETL), EL-16-40-TC from Optotune, to solve the TIE for obtaining the phase distributions at the output plane. The ETL is inserted at the Fourier plane of the first lens. In this setup, the magnification ratio is fixed even when the object is located at different depth positions. The ETL is basically a variable focal length lens^8^^,^^10^ that is used to implement nonmovable TIE.^17^ After solving TIE by using these images, the phase distribution can be retrieved.^16^^–^^22^

It is well known that the light emitted by a fluorescence sample is not coherent. However, in the applications of biological samples, fluorescence proteins are stained to the cells, and then the fluorescence distribution of nucleus is assumed as a quasi-point-like monochromatic source, because its size is about 10  μm. Besides, in our optical system, a band-pass filter with a central wavelength of 575 nm and width of 10 nm is used to increase the temporal coherence. Therefore, partial spatial coherence of the fluorescence light can be considered after propagation through the optical system even if there is no coded aperture.^13^ For the partial coherence field, the generalized definition of TIE for phase has been discussed in Refs. 15 and 16 and it has been demonstrated in several studies to obtain phase distributions.^17^^–^^19^ In case of partial coherence field, the TIE can be derived from the Helmholtz equation, which is explained as follows:

$$\nabla .[I_{z}(x,y)\times \nabla \phi_{z}(x,y)]=-\frac{2\pi }{\lambda }\frac{\partial I_{z}(x,y)}{\partial z},$$ (1)

where λ, $I_{z}(x,y)$, and $\phi_{z}(x,y)$, are the wavelength, intensity distributions, and phase distributions at depth z, respectively. The symbol ∇ is the gradient operator in the transverse plane (x,y). The TIE for the phase is solved by using Fourier transform (FT).^16^^–^^22^ Here, we consider the intensity distribution, $I_{0}(x,y)$, and defocus intensity distributions can be captured, which are to be located at axial positions z=0 and z=±Δz, respectively. The obtained phase can be determined by the following equation:

$$\phi_{0}(x,y)=-\frac{2\pi }{\lambda }{\mathrm{FT}}^{-1}[\frac{1}{4\pi^{2}(u^{2}+v^{2})}\mathrm{FT}[\nabla .\frac{\nabla }{I_{0}(x,y)}\times {\mathrm{FT}}^{-1}\{\frac{1}{4\pi^{2}(u^{2}+v^{2})}\times \mathrm{FT}\{\frac{\partial I_{0}(x,y)}{\partial z}\}\}]].$$ (2)

In the previous equation, u and v are the spatial frequencies corresponding to x and y coordinates, respectively. The intensity derivative used in Eq. (2) is calculated approximately by using the difference between the two intensity distributions obtained at depth positions Δz and −Δz, as is shown in the following equation:

$$\frac{\partial I_{0}(x,y)}{\partial z}=\frac{I_{\Delta z}(x,y)-I_{-\Delta z}(x,y)}{2\Delta z}.$$ (3)

Hence, the phase distribution can be retrieved by combining Eqs. (2) and (3). It should be noted that the intensity image $I_{0}(x,y)$ can also be a defocused version of the input object. Yet, our method allows to record the light field corresponding to the selected axial distance with the TIE-based algorithm. However, in the original TIE method, focus image and in- and out-of-focus images are always used to get phase information.

Now the complex amplitude, H(x,y), is obtained by multiplying the square root of the intensity image, $I_{0}(x,y)$, with the retrieved phase, $\phi_{0}(x,y)$, and this complex amplitude is Fresnel propagated. From the back- or forward-propagated distribution, intensity images of the fluorescent samples can be estimated at multiple planes located at different distances.^21^^,^^22^

## 3 Experiments and Results

Various experiments were performed to verify the proposed method for 3-D fluorescence imaging using the optical setup shown in Fig. 1. Microscope objective of 20× magnification was used for imaging fluorescent microbeads and a biological sample. In the first experiment, single plane fluorescence imaging of microbeads of 10.4  μm diameter is verified and the focusing capability is demonstrated. A blue LED of 470-nm wavelength was used as an excitation light source for the microbeads. The fluorescent microbeads emit light with a wavelength ranging from 550 to 600 nm. A bandpass filter centered at 575 nm with a bandwidth of 10 nm was used in front of the image sensor. The effective pixel number of the image sensor is 700×700  pixels with a pixel pitch of 4.54  μm.

**Fig. 2:**
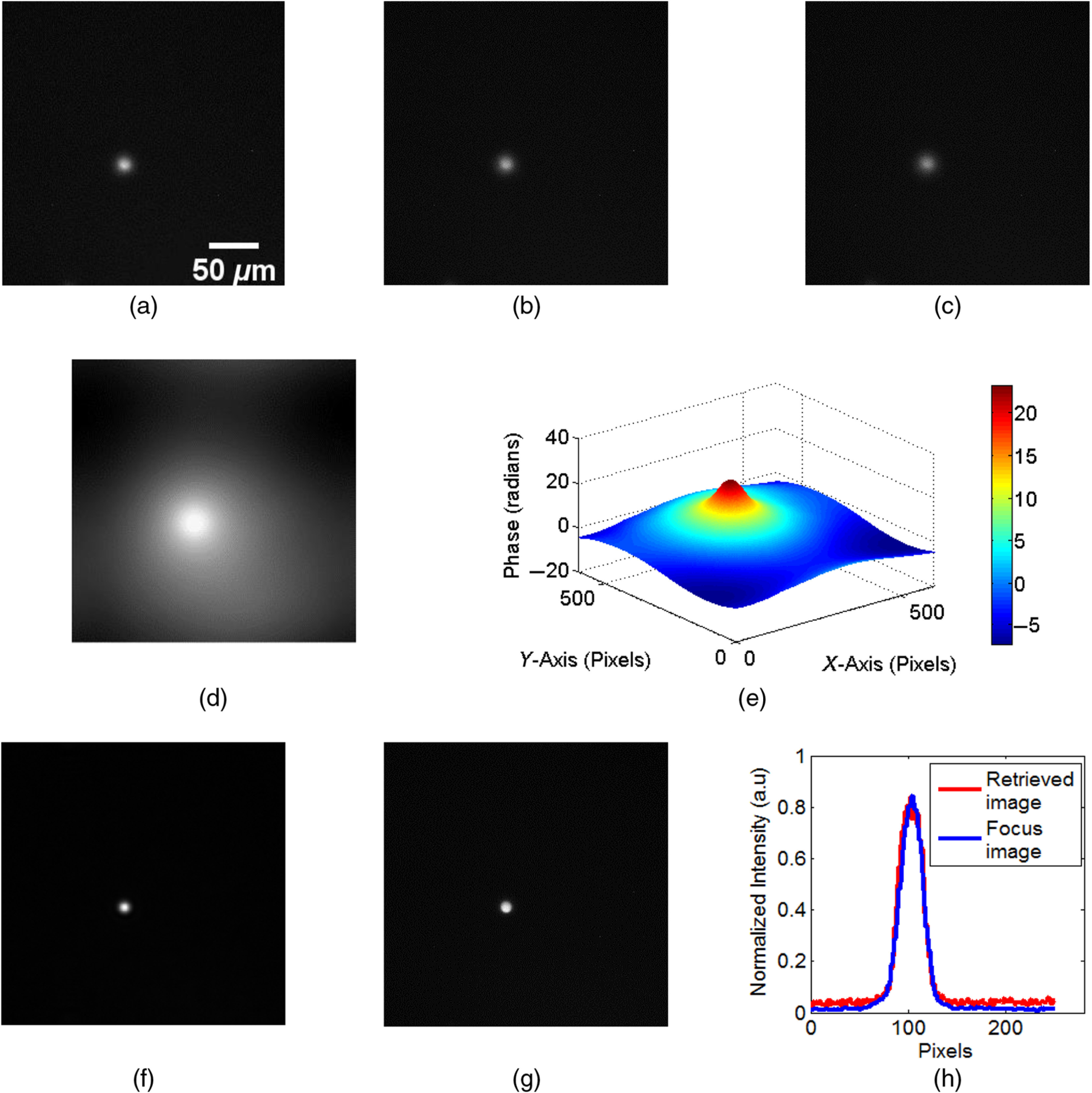
Experimental results for single plane imaging. (a)–(c) Three defocus intensity images, (d) the reconstructed phase image, (e) surface plot of phase, (f) recovered focus image, (g) original focus image recorded in the experiment, and (h) line plot on the recovered and original focus image.

Results of single plane fluorescence imaging are shown in Fig. 2. Three defocus intensity images of fluorescent microbeads were recorded, as shown in Figs. 2(a)–2(c), by changing the focal length of the ETL. A focus intensity image of the input plane was also recorded to compare it with the retrieved focused fluorescent image obtained with the proposed method. The defocus distances of the three recorded images from the ideal focus image are 20, 30, and 40  μm, respectively. From these images, the phase image corresponding to the intensity image shown in Fig. 2(b) was retrieved by solving the TIE. The result is shown in Fig. 2(d). Here, the image shown in Fig. 2(b) is treated as the intensity distribution $I_{0}$, as used in Eqs. (2) and (3), and the images shown in Figs. 2(a) and 2(c) are considered as $I_{\Delta z}$ and $I_{-\Delta z}$, respectively, for solving the TIE. Figure 2(e) shows the surface plot of the retrieved phase. The focus image, shown in Fig. 2(f), was recovered after backpropagation of the complex function calculated from the amplitude image, shown in Fig. 2(b), and the corresponding retrieved phase. The original focus image recorded in the experiment is shown in Fig. 2(g). Figure 2(h) shows the line plot across the image of the bead on the recovered and the original focus images. Here, the reconstruction distance from central defocus image is 24 mm. From these results, it can be seen that the focus image can be obtained successfully from the three defocus images, which proves the capability of our method to provide 3-D fluorescence imaging.

**Fig. 3:**
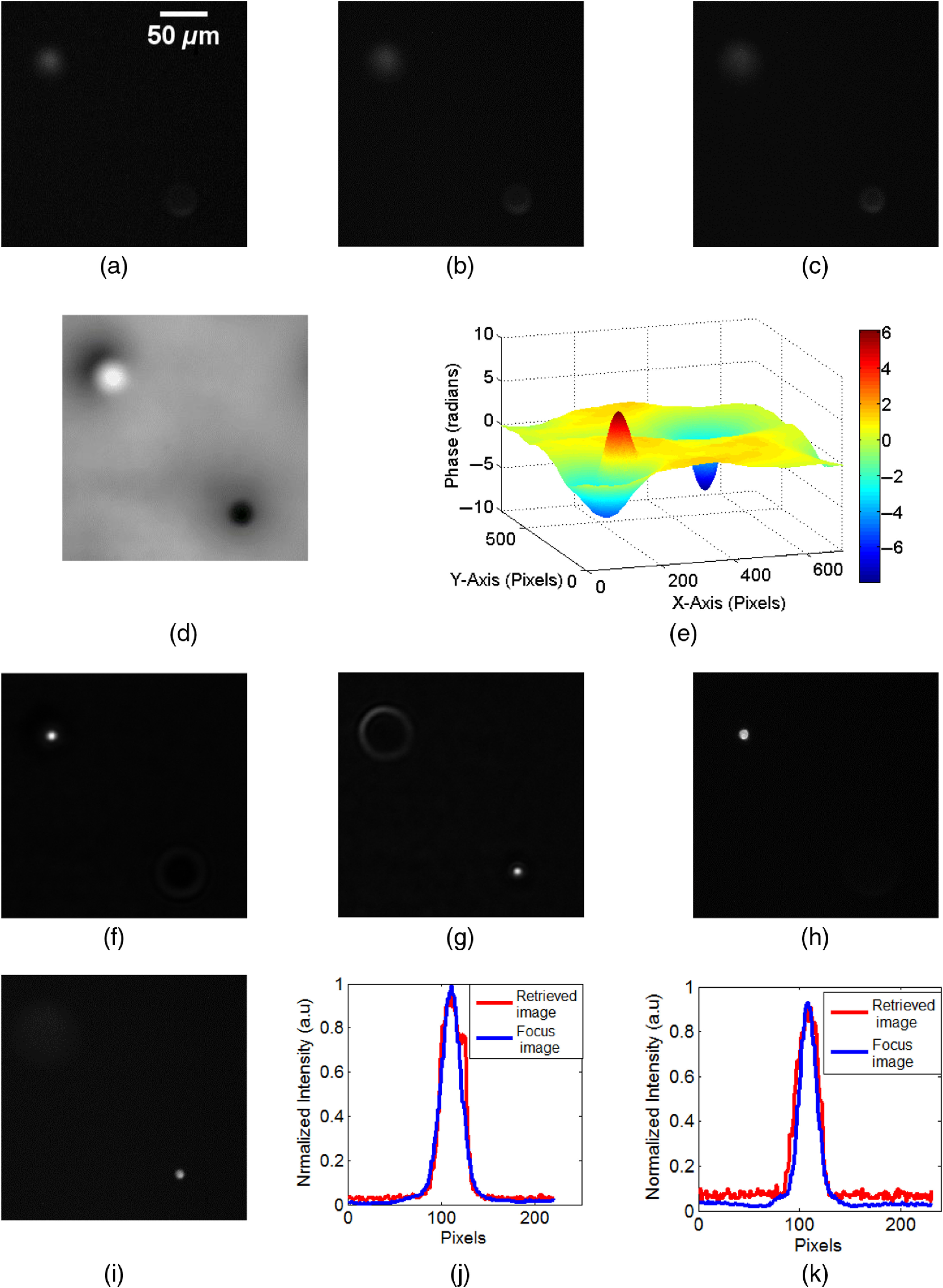
Experimental results for double plane imaging. (a)–(c) Three defocus intensity images, (d) the reconstructed phase image using TIE-based phase retrieval, and (e) surface plot of the phase in (d). Recovered focus images in which (f) one bead is at focus and (g) second bead is at focus. Original focus images recorded in a conventional imaging experiment, where (h) one of the beads is at focus and (i) the second bead is at focus. (j), (k) Line plots of the intensity along the position of the beads on the recovered and the original image for (j) the first focusing distance and (k) the second focusing distance.

Another experiment was performed to demonstrate the 3-D fluorescence imaging capability of the proposed method. For this experiment, two layers of fluorescent beads were prepared on glass slides with a separation of more than 80  μm and again, three defocus images were recorded, as shown in Figs. 3(a)–3(c). In this case, defocus images were recorded in between with an interval of 5  μm. The phase image shown in Fig. 3(d) corresponds to the phase associated with the intensity image in Fig. 3(b). This image in Fig. 3(b) is considered as $I_{0}$; the images shown in Figs. 3(a) and 3(c), as $I_{\Delta z}$ and $I_{-\Delta z}$, respectively. Figure 3(e) shows the surface plot of the phase distribution in Fig. 3(d). Figure 3(e) indicates that the defocused image to obtain the complex amplitude distribution is located at a farther position than that of Fig. 2(e). The maximum phase amount depends on the distance from the observed defocused plane to the focused plane to be reconstructed. The effect of two beads located at different distances is clearly noticeable. The complex light field is determined from the retrieved phase and its corresponding intensity image. Images focused at different planes, showing clear intensity images of the beads, were recovered by Fresnel propagation of the complex function. These recovered focus images are shown in Figs. 3(f) and 3(g). The corresponding original images, with beads in focus, recorded experimentally by conventional imaging, are shown in Figs. 3(h) and 3(i). Reconstruction distance from the central defocus image for the upper bead is 45 mm to positive side and 36 mm for other beads to the negative side from the measurement plane. Figures 3(j) and 3(k) show line plot across the images of beads of the recovered and original intensity distributions in both planes. From these results, it can be confirmed that the 3-D fluorescence imaging can be performed by our proposed method.

**Fig. 4:**
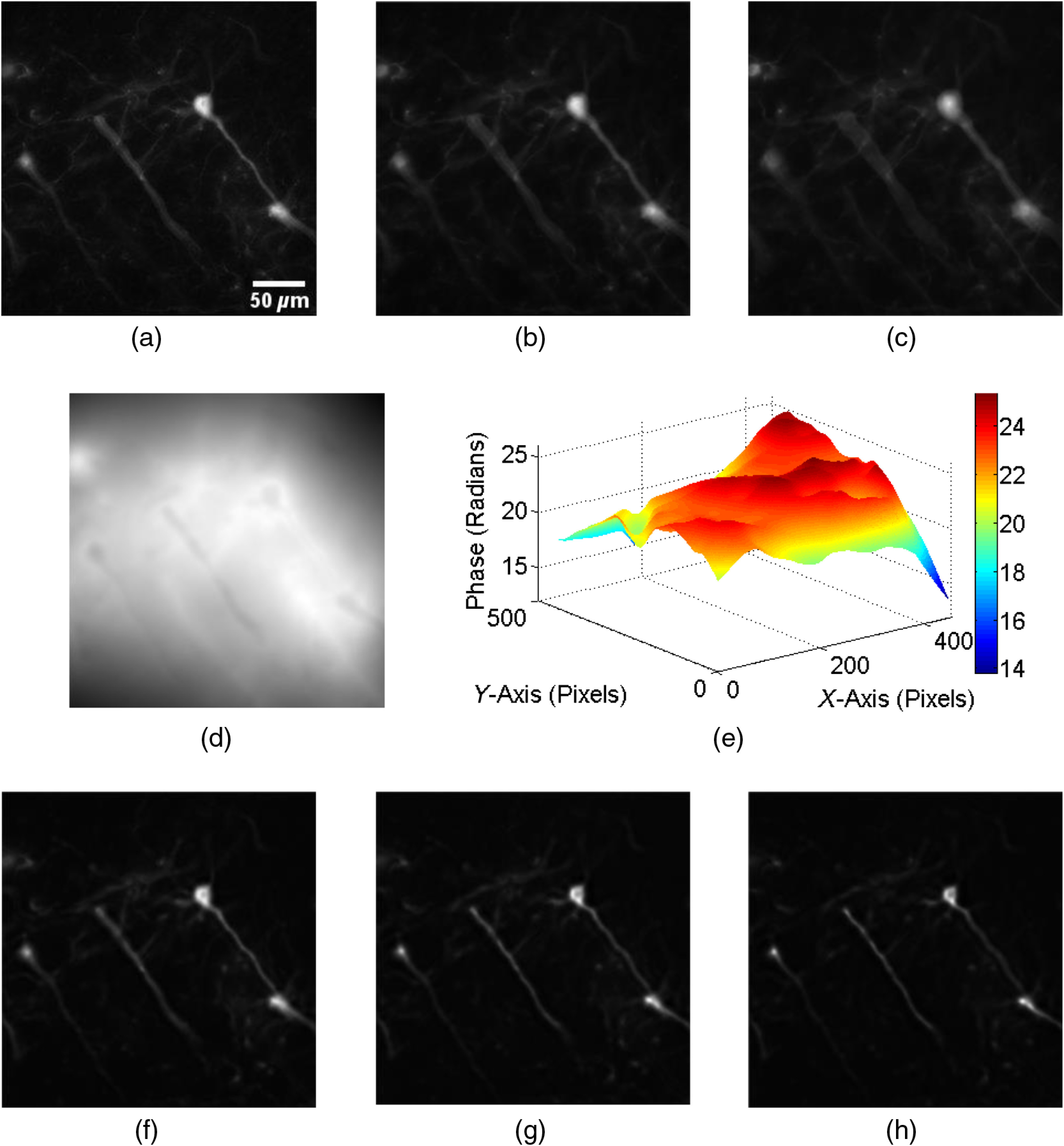
Experimental results for fluorescence from neurite and cells. (a)–(c) Three defocus intensity images; (d) reconstructed phase image; (e) surface plot of phase in (d); (f)–(h) recovered intensity images at distances of 22, 26, and 30 mm, respectively, from measurement plane.

In the third experiment, the 3-D imaging capability of the proposed method was verified on a biological sample. GCaMP-expressing neurons in a cerebral slice of 100  μm thickness from a CaMK2-CreERT2/Rosa-GCaMP3^23^ mouse treated with tamoxifen were visualized by anti-GFP immunostaining with Cy3-tyramine signal amplification system. For this sample, a green LED of the central wavelength of 575 nm was used as the light source to excite the fluorescent-labeled neurite and cells. The fluorescent-labeled neurite and cells emit light with a wavelength ranging from 620 to 640 nm, thus a bandpass filter centered at 630 nm was used in front of the image sensor. The three experimentally recorded defocus images of the biological sample are shown in Figs. 4(a)–4(c). Fluorescent light from neurite can be clearly seen. These defocus images were recorded with an interval of 2  μm. Here, the sample is shifted along the depth direction by a translation stage to obtain the defocused images instead of ETL. The retrieved phase obtained by TIE algorithm corresponding to the intensity images in Figs. 4(a)–4(c) is shown in Fig. 4(d). The surface plot of the phase is shown in Fig. 4(e). From the complex field retrieved from the phase in Fig. 4(d) and intensity in Fig. 4(b), reconstructed images in the image plane at different depths of 22, 26, and 30 mm can be recovered, which are shown in Figs. 4(f)–4(h), respectively. From these results, it can be concluded that with our proposed technique, it is possible to perform 3-D fluorescence imaging of biological sample in multiple planes from defocused intensity images.

## 4 Conclusion

In this work, we have presented a scanless 3-D fluorescence imaging scheme based on the TIE technique that uses the Fresnel backpropagation of a complex wave function calculated from the phase retrieved by TIE and its corresponding intensity image. In the proposed imaging technique, the phase distribution corresponding to defocus fluorescence images is retrieved by a phase retrieval algorithm based on TIE. Images focused at different planes can be estimated from the complex distribution after the numerical calculation of inverse Fresnel propagation. Through the proposed imaging approach, it is straightforward to perform 3-D fluorescence imaging in multiple planes. Real-time imaging, for example, for live-cell imaging will be also possible because intensity fluorescence images can be captured with the help of fast ETLs and focus images are retrieved by fast FT-based numerical methods. The feasibility of the proposed 3-D fluorescence imaging method is demonstrated by experimental results obtained from distributions of microbeads and a biological sample.
